# Preserved Microvascular Endothelial Function in Young, Obese Adults with Functional Loss of Nitric Oxide Signaling

**DOI:** 10.3389/fphys.2015.00387

**Published:** 2015-12-22

**Authors:** John W. Harrell, Rebecca E. Johansson, Trent D. Evans, Joshua J. Sebranek, Benjamin J. Walker, Marlowe W. Eldridge, Ronald C. Serlin, William G. Schrage

**Affiliations:** ^1^Bruno Balke Biodynamics Laboratory, Department of Kinesiology, University of Wisconsin–MadisonMadison, WI, USA; ^2^Department of Anesthesiology, University of Wisconsin Hospital and Clinics, University of Wisconsin–MadisonMadison, WI, USA; ^3^The John Rankin Laboratory of Pulmonary Medicine, Department of Population Health Sciences, School of Medicine and Public Health, University of Wisconsin–MadisonMadison, WI, USA; ^4^Department of Pediatrics, University of Wisconsin Hospital and Clinics, University of Wisconsin–MadisonMadison, WI, USA; ^5^Department of Educational Psychology, University of Wisconsin–MadisonMadison, WI, USA

**Keywords:** obesity, microcirculation, endothelium, nitric oxide synthase, vascular function

## Abstract

Data indicate endothelium-dependent dilation (EDD) may be preserved in the skeletal muscle microcirculation of young, obese adults. Preserved EDD might be mediated by compensatory mechanisms, impeding insight into preclinical vascular dysfunction. We aimed to determine the functional roles of nitric oxide synthase (NOS) and cyclooxygenase (COX) toward EDD in younger obese adults. We first hypothesized EDD would be preserved in young, obese adults. Further, we hypothesized a reduced contribution of NOS in young, obese adults would be replaced by increased COX signaling. Microvascular EDD was assessed with Doppler ultrasound and brachial artery infusion of acetylcholine (ACh) in younger (27 ± 1 year) obese (*n* = 29) and lean (*n* = 46) humans. Individual and combined contributions of NOS and COX were examined with intra-arterial infusions of *l*-NMMA and ketorolac, respectively. Vasodilation was quantified as an increase in forearm vascular conductance (ΔFVC). Arterial endothelial cell biopsies were analyzed for protein expression of endothelial nitric oxide synthase (eNOS). ΔFVC to ACh was similar between groups. After *l*-NMMA, ΔFVC to ACh was greater in obese adults (*p* < 0.05). There were no group differences in ΔFVC to ACh with ketorolac. With combined NOS-COX inhibition, ΔFVC was greater in obese adults at the intermediate dose of ACh. Surprisingly, arterial endothelial cell eNOS and phosphorylated eNOS were similar between groups. Younger obese adults exhibit preserved EDD and eNOS expression despite functional dissociation of NOS-mediated vasodilation and similar COX signaling. Compensatory NOS- and COX-independent vasodilatory mechanisms conceal reduced NOS contributions in otherwise healthy obese adults early in life, which may contribute to vascular dysfunction.

## Introduction

While it is clear obese adults in middle age and beyond exhibit poor vascular function leading to overt cardiovascular disease, the etiology of preclinical vascular changes in younger obese adults remains uncertain. This knowledge holds clinical relevance because individuals who are obese as adolescents are unlikely to attain a healthy weight (Fildes et al., 2015), as up to 63% will remain obese into adulthood (Serdula et al., 1993). The association between obesity and increased risk of cardiovascular disease is well established in older adults, and vascular impairments are considered to be an early marker of disease onset (Davignon and Ganz, 2004; Landmesser et al., 2004). Early awareness of mechanistic changes in endothelium-dependent dilation (EDD) is paramount for starting interventions aimed at restoring lost mechanisms or leveraging compensatory mechanisms to preserve EDD and prevent development of cardiovascular disease.

The effect of obesity on EDD in the absence of aging remains unclear. Discrepancies in data likely stem from differences in the age of subjects, limb differences, and presence of co-morbidities such as hypertension. Impaired EDD has been observed in conduit arteries of older (>65 year) obese adults (Acree et al., 2007) and in the microvasculature of middle-aged (>40 year) obese adults (Perticone et al., 2001; Van Guilder et al., 2008). Steinberg et al. reported decreased EDD in the leg of obese adults (~35 year; Steinberg et al., 1996), whereas we recently reported preserved EDD in the forearm of obese adults (~33 year; Limberg et al., 2013) that has yet to be confirmed.

The primary vascular control mechanisms responsible for eliciting EDD appear compromised in humans at increased risk of cardiovascular disease. Older (Taddei et al., 2001) and hypertensive (Taddei et al., 1998) adults exhibit reduced contribution of nitric oxide synthase (NOS) and restraint of EDD by cyclooxygenase (COX). On the other hand, NOS and COX can work in a compensatory fashion, where reductions in one signal can increase the other (Osanai et al., 2000; Dinenno and Joyner, 2004). Further, if NOS is uncoupled or not functioning, vasodilation may rely more heavily on an alternate vasodilatory mechanism (e.g., cytochrome P450; Durand and Gutterman, 2013; Spilk et al., 2013). Given the pathologic progression of obesity toward overt cardiovascular disease, EDD may appear preserved in younger obese adults (Limberg et al., 2013) while vascular signaling mechanisms can be subtly altered decades prior to development of clinical disease.

We studied a substantial number of carefully selected young adults using invasive approaches and pharmacological tools because small preclinical vascular changes are challenging to detect. We aimed to confirm earlier results of intact microvascular EDD in younger obese adults, and directly test vasodilatory mechanisms potentially masking vascular dysfunction. Forearm microvascular function was tested in young (<40 years) lean and obese adults matched for age and physical activity, in the absence of confounding clinically relevant cardiovascular risk factors, such as age, hypertension, diabetes, or dyslipidemia. We hypothesized EDD would be preserved in young, obese adults due to a shift in the balance of NOS and COX signaling mediating EDD. Hypothesized results would be consistent with the concept of young obese adults exhibiting preserved endothelial function with early mechanistic changes contributing to the development of obesity-induced cardiovascular disease.

## Materials and methods

### Subjects

Seventy-five subjects participated in this study (lean *n* = 46, obese *n* = 29). Subjects were younger (18–40 year), healthy, physically inactive (<60 min per week), non-smokers, and not taking cardiovascular medications. Obesity was defined as a body mass index (BMI) ≥ 30 kg m^−2^ or a waist circumference > 102 cm (males) or > 88 cm (females). Healthy controls were lean (BMI < 25 kg m^−2^). Female subjects were not pregnant (urine test) and studied on days 1–5 of the menstrual cycle to minimize effects of female hormones. Females on hormonal contraception (lean *n* = 13, obese *n* = 10) were studied during the placebo phase. Subjects were instructed to refrain from caffeine, exercise, non-steroidal anti-inflammatory drugs, and alcohol for 24 h prior to the study. All subjects provided written informed consent. Study procedures were approved by the Institutional Review Board at the University of Wisconsin-Madison, and obeyed the standards of the Declaration of Helsinki.

### Measurements

Height and weight were measured for calculation of BMI (kg m^−2^) and waist circumference was measured. Dual-energy x-ray absorptiometry (GE Lunar Prodigy; Milwaukee, WI) measured body composition, forearm mass, and lean forearm mass. Blood was collected following a 12-h fast for measurement of glucose and lipids. Plasma was stored at −80°C and later analyzed for insulin and leptin (Millipore; Billerica, MA, USA).

### Brachial artery catheterization

Following local anesthesia (2% lidocaine), a 20-gauge catheter was inserted in the brachial artery of the non-dominant forearm (antecubital fossa) under aseptic conditions. The catheter was used for blood sampling, local infusion of drugs, arterial endothelial cell biopsies, and blood pressure measurements.

### Blood flow

Doppler Ultrasound (Vivid 7, General Electric) measured brachial artery blood velocity and diameter for calculation of forearm blood flow (FBF). The 12 MHz linear array probe was placed over the brachial artery with an insonation angle ≤ 60° and the sample volume adjusted to include the width of the brachial artery (Limberg et al., 2013). The angle-corrected, intensity-weighted Doppler audio information from the GE Vivid ultrasound was processed into a velocity signal by a custom interface unit via Fourier transform with a calibrated scale (Herr et al., 2010) and sampled in real time at 400 Hz (PowerLab, ADInstruments). Brachial artery diameter was measured as reported previously (Limberg et al., 2013). FBF was calculated as the product of vessel cross sectional area (CSA, cm^2^) and mean blood velocity (MBV, cm s^−1^) and is reported in mL min^−1^ [FBF = (MBV) (CSA) (60 s min^−1^)].

### Drug infusions

Pharmaceuticals were mixed for each study visit to standard concentrations [*Acetylcholine* (ACh), Bausch and Lomb; *Nitroprusside* (NTP), Hospira, Inc.; *l-N*^*G*^*-monomethyl arginine citrate* (*l*-NMMA), Clinalfa; *ketorolac* (Keto*)*, Wockhardt]. ACh assessed endothelial function (1, 4, and 16 μg 100 g^−1^ min^−1^) and NTP tested vascular smooth muscle function (0.5, 1, and 2 μg 100 g^−1^ min^−1^; Taddei et al., 2001; Schrage et al., 2005). *l*-NMMA inhibited NOS (loading dose: 10 mg min^−1^ for 5 min, maintenance dose: 1 mg min^−1^; Dinenno and Joyner, 2003, 2004) and Keto inhibited COX (loading dose: 1.2 mg min^−1^ for 5 min, maintenance dose: 0.1 mg min^−1^; Dinenno and Joyner, 2004; Schrage et al., 2004). ACh and NTP infusions were adjusted for lean forearm mass to minimize systemic responses, to standardize drug concentrations across conditions, and to control for differences in lean forearm size between subjects. Finally, the majority of drug was delivered to muscle tissue as muscle blood flow is at least 2.5–5 times greater than adipose tissue (Delp et al., 1998) and adipose tissue blood flow remains relatively constant under resting conditions (Heinonen et al., 2012).

### Study protocol

Five study conditions were conducted in the supine position. ACh and NTP were infused in randomized and counterbalanced order at three increasing doses for 3 min each. Trials were separated by 10 min to allow baseline hemodynamics to return (Kirby et al., 2009; Limberg et al., 2013). After control ACh and NTP infusions, the loading dose of *l*-NMMA or Keto (random order) was infused over 5 min and followed by the maintenance dose for the remainder of the study visit. A second ACh trial was conducted with *l*-NMMA or Keto infusion. Then, the loading dose of the second inhibitor was infused over 5 min followed by the maintenance dose to induce combined inhibition. Finally, a third ACh and second NTP trial were performed under combined *l*-NMMA and Keto infusion.

### Arterial endothelial biopsy

In a subset of subjects, prior to drug infusions, arterial endothelial cells (ECs) were harvested from the brachial artery using 0.018 inch diameter J-shaped wires introduced via the arterial catheter (Colombo et al., 2002; Donato et al., 2007; Silver et al., 2007; Pierce et al., 2009). The distal portion of the wire was transferred to a conical tube containing dissociation buffer (0.5% bovine serum albumin, 2 mM EDTA, and 100 μg ml^−1^ heparin in PBS, pH 7.4) kept at 4°C. After rinsing 10 min with dissociation buffer, ECs were recovered by centrifugation and fixed with 3.7% formaldehyde in PBS for 10 min. Cells were washed, transferred to slides (Sigma), air-dried at 37°C, and stored at −80°C until immunofluorescent staining. Cells were rehydrated and non-specific binding sites were blocked with 5% donkey serum. Cells were first incubated with primary antibody against endothelial nitric oxide synthase (eNOS, BD Transduction) or phosphorylated-eNOS S1177 (p-eNOS, Abcam) followed sequentially by Alexaflour 555 fluorescent secondary antibody (Life Technologies), Von-Willebrand Factor primary antibody (VWF, Abcam), and Alexaflour 488 fluorescent secondary antibody (Life Technologies). For analysis, slides were viewed with an epifluorescence microscope (Zeiss). Cell images (≥14 and >20 images for most subjects) were captured by a digital camera. ECs were identified by positive staining for VWF and nuclear integrity was confirmed with DAPI (4′,6′-diamidino-2-phenylindole). Protein expression in cells was quantified as background-corrected average pixel intensity (Adobe Photoshop CS5) and normalized to a concurrently stained human umbilical vein endothelial cell (HUVEC) slide to minimize potential confounding effects of differences in staining intensity across staining sessions (Colombo et al., 2002; Donato et al., 2007; Silver et al., 2007; Pierce et al., 2009). Technicians were blinded to subject identity during staining and analysis.

### Data acquisition and analysis

Blood pressure and heart rate (ECG; Datex-Ohmeda) were digitized throughout each trial at 400 Hz (PowerLab, ADinstruments). Data were analyzed off line using LabChart7 software to yield average arterial blood pressure, heart rate, and MBV during the last 30 s of rest and each drug dose (MBV used for FBF calculation, see Section Blood Flow). FBF (mL min^−1^) calculations were normalized for mean arterial blood pressure (MAP) as forearm vascular conductance [FVC (mL min^−1^ 100 mmHg^−1^) = FBF/MAP] to account for group differences in blood pressure (Table 1).

**Table 1:** **Subject characteristics**.

	**Lean**	**Obese**
*n* (M/F)	48(24∕24)	29(10∕19)
Age (year)	26±6	29±7
Height (cm)	172±9	171±11
Weight (kg)	66±8	106±22^*^
Waist (cm)	78±6	111±14^*^
BMI (kg m^−2^)	22±2	36±6^*^
Body fat (%)	27±9	48±10^*^
Forearm mass (g)	882±195	1240±288^*^
Lean forearm mass (g)	785±214	856±243
MAP (mmHg)	83±11	88±12^*^
Total cholesterol (mg dL^−1^)	154±32	159±29
LDL (mg dL^−1^)	86±21	93±23
HDL (mg dL^−1^)	55±17	46±13^*^
Triglycerides (mg dL^−1^)	74±27	94±32^*^
Glucose (mg dL^−1^)	70±9	72±8
Insulin (μU mL^−1^)	9±3	18±9^*^
PAQ (kcal wk^−1^)	1273±897	1142±908

*Data are presented as mean ± SE. M, male; F, female; BMI, body mass index; MAP, mean arterial blood pressure; LDL, low-density lipoprotein; HDL, high-density lipoprotein; PAQ, physical activity questionnaire score*.

* *p < 0.05 vs. Lean*.

### Statistical analysis

Statistical analysis was performed using Minitab Version 16 (Minitab, Inc.). The main dependent variable was a change in FVC in response to drug infusions (ΔFVC = FVC_Infusion_ – FVC_Baseline_). ΔFVC accounts for baseline FVC differences resulting from group differences in resting blood pressure and/or the effect of inhibitors on blood pressure. Primary analysis was a non-parametric ANCOVA to investigate the between-group differences in ΔFVC at each ACh dose without and with inhibitors (*l*-NMMA, Keto, or combined inhibition). In close consult with our collaborating biostatistician (coauthor RC Serlin), an ANCOVA was used to compare ΔFVC between groups at each NTP dose with and without combined inhibition. Group comparisons at each dose of ACh and NTP provide the greatest amount of power to detect group differences, and minimize Type I error rate. Another major advantage of the ANCOVA is the elimination of irrelevant comparisons imbedded in an ANOVA (e.g., high dose ACh in leans vs. low dose ACh + *l*-NMMA in obese). A Kruskal-Wallis test investigated the effect of blockade order (*l*-NMMA first vs. Keto first) on ΔFVC in response to ACh with combined inhibition. Mann-Whitney tests determined the significant effect of group on subject characteristics and eNOS and p-eNOS protein expression. Significance was determined *a priori* at *p* < 0.05. Data are presented as mean (standard deviation).

## Results

Subject characteristics of 46 lean controls and 29 obese adults are summarized in Table 1. Groups were well matched for age and physical activity. By design, obese adults exhibited significantly greater weight, waist circumference, BMI, and percent body fat (*p* < 0.05). Obese adults displayed greater forearm mass (*p* < 0.05), but similar lean forearm mass. Further, the obese adults displayed higher MAP, higher triglycerides, and lower HDL (*p* < 0.05), though all were within clinically healthy ranges. Despite similar blood glucose, total cholesterol, and LDL cholesterol between groups, obese adults exhibited higher insulin concentration (*p* < 0.05). Collectively, subject selection criteria controlled for many common cardiovascular risk factors, allowing the study to focus on the primary impact of obesity on skeletal muscle EDD.

### Endothelium-dependent dilation

**Figure 1:**
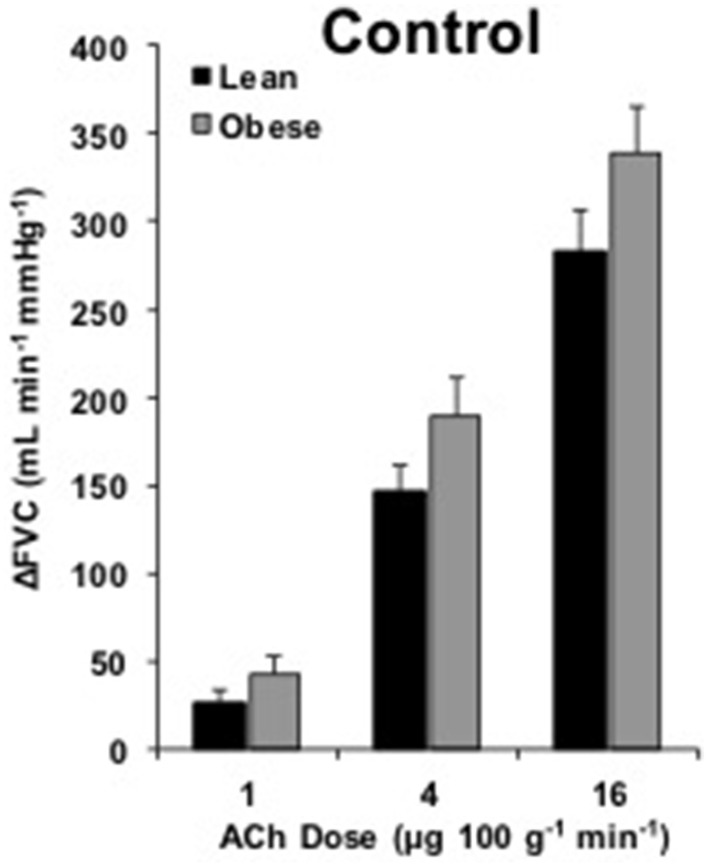
**ACh responses**. Change in forearm vascular conductance (ΔFVC) with intra-arterial ACh infusion. ΔFVC without inhibition was not different between lean (*n* = 46) and obese (*n* = 29) adults.

**Figure 2:**
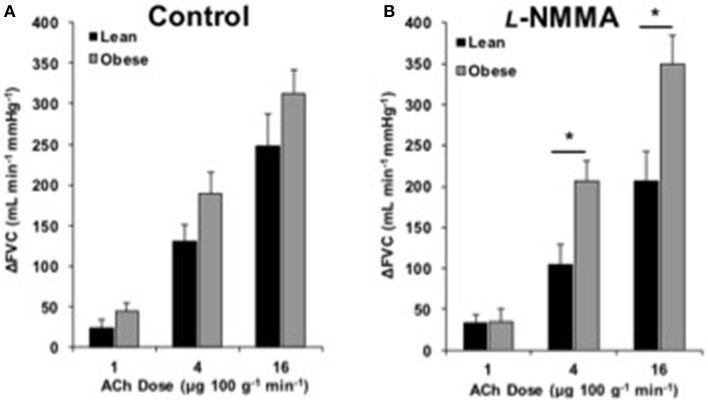
**ACh responses with l-NMMA**. Change in forearm vascular conductance (ΔFVC) with intra-arterial ACh infusion and NOS inhibition (*l*-NMMA). **(A)** ΔFVC without inhibition was not different between lean (*n* = 18) and obese (*n* = 21) adults. **(B)** ΔFVC with *l*-NMMA was greater in obese adults (*n* = 21) than lean adults (*n* = 18) at 4 and 16 μg 100 g^−1^ min^−1^. ^*^Significant group difference, *p* < 0.05.

**Figure 3:**
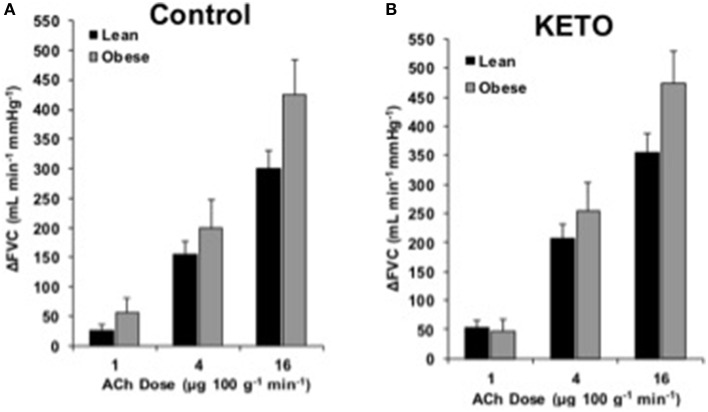
**ACh responses with Keto**. Change in forearm vascular conductance (ΔFVC) with intra-arterial ACh infusion and cyclooxygenase inhibition (Keto). **(A)** ΔFVC was not different between lean (*n* = 26) and obese (*n* = 7) adults. **(B)** ΔFVC with Keto was not different between lean (*n* = 26) and obese (*n* = 7) adults.

**Figure 4:**
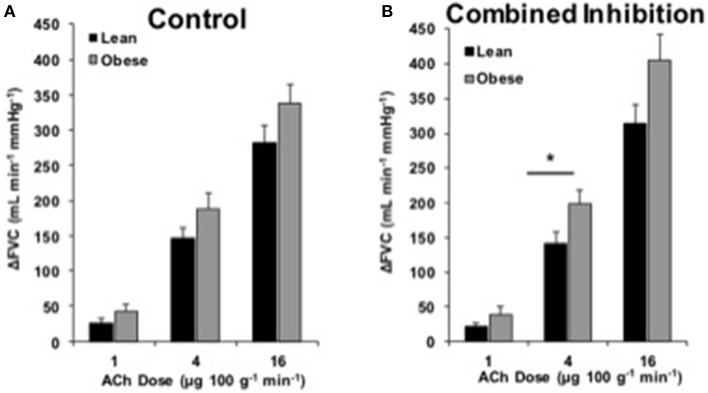
**ACh responses with combined inhibition**. Change in forearm vascular conductance (ΔFVC) with intra-arterial ACh infusion and combined NOS-COX inhibition (*l*-NMMA and Keto). **(A)** ΔFVC was not different between lean (*n* = 46) and obese (*n* = 29) adults (same as Figure 1). **(B)** ΔFVC with combined NOS-COX inhibition was greater in obese adults (*n* = 29) than lean adults (*n* = 46) at 4 μg100^−1^ g min^−1^. ^*^Significant group difference, *p* < 0.05.

FBF and FVC responses to ACh are summarized in Supplementary Table 1. The increase in FVC (ΔFVC) with ACh was not different between groups at any dose (Figure 1). With *l*-NMMA, ΔFVC was lower in lean adults at the 4 and 16 μg 100 g^−1^ min^−1^ doses (Figure 2B, *p* < 0.05). With Keto, ΔFVC was similar between groups at all doses (Figure 3B). ΔFVC with combined NOS-COX inhibition was greater in the obese adults at the 4 μg 100 g^−1^ min^−1^ dose (Figure 4B, *p* < 0.05), and the difference was quantitatively similar at 16 μg 100 g^−1^ min^−1^, but did not achieve statistical significance. The order of inhibition (*l*-NMMA first vs. Keto first) did not impact ΔFVC during combined NOS-COX inhibition. Analysis of ΔFBF resulted in similar conclusions (data not shown).

MAP values for both groups during ACh trials are presented in Supplementary Table 1. Across most trials, ACh infusion decreased MAP (1–4 mmHg) in both groups (*p* < 0.05). *l*-NMMA increased baseline MAP only in lean subjects (*p* < 0.05). Keto did not change baseline MAP in either group. Combined NOS-COX inhibition increased baseline MAP in both groups during ACh trials (*p* < 0.05).

### Endothelium-independent dilation

**Figure 5:**
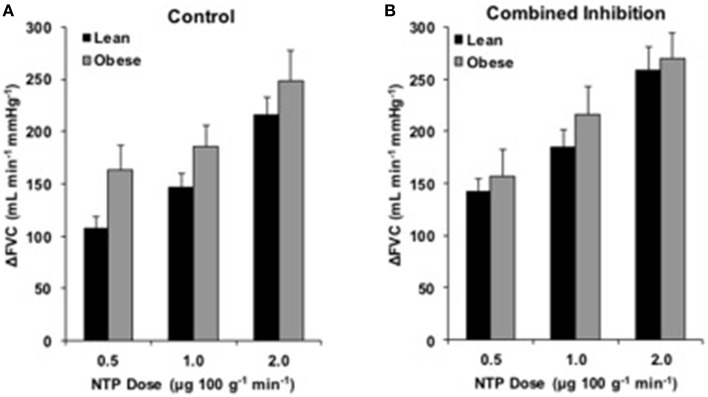
**NTP responses with combined inhibition**. Change in forearm vascular conductance (ΔFVC) with intra-arterial NTP infusion. **(A)** ΔFVC without inhibition was not different between obese (*n* = 26) and lean (*n* = 44) adults. **(B)** ΔFVC with combined NOS-COX inhibition was not different between obese adults (*n* = 19) and lean adults (*n* = 36).

Endothelium-independent vascular responses to NTP are listed in Supplementary Table 2. ΔFVC was not different between groups at any dose (Figure 5A). Combined NOS-COX inhibition did not alter ΔFVC responses (Figure 5B). Analysis of ΔFBF revealed no group differences, similar to ΔFVC (data not shown).

MAP values for both groups during NTP infusions are listed in Supplementary Table 2. In general, NTP infusion decreased MAP (5–8 mmHg) in both groups (*p* < 0.05). Combined NOS-COX inhibition increased baseline MAP only in lean subjects during NTP trials (*p* < 0.05).

### Endothelial cell biopsy

**Figure 6:**
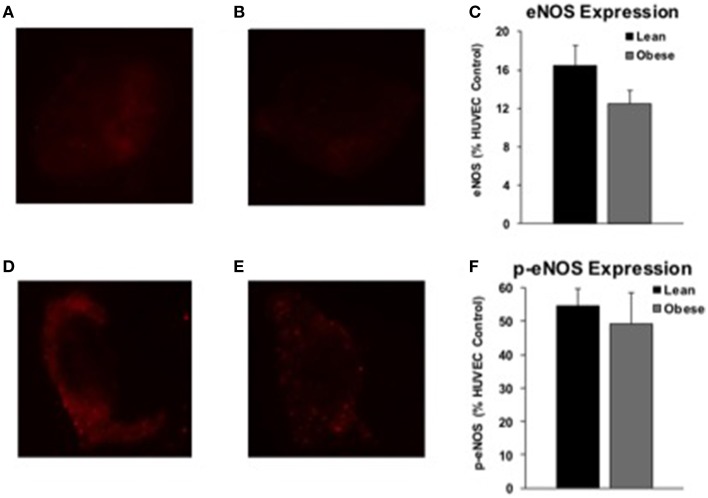
**eNOS and p-eNOS expression**. Endothelial NOS (eNOS) and phosphorylated eNOS (p-eNOS) protein expression from arterial cell biopsies. Representative images of arterial eNOS staining from a lean **(A)** and obese **(B)** subject. eNOS protein expression was similar between a subset of lean (*n* = 14) and obese (*n* = 7) adults **(C)**. Representative images of arterial p-eNOS staining from a lean **(D)** and obese **(E)** subject. p-eNOS protein expression was similar between a subset of lean (*n* = 16) and obese (*n* = 5) adults **(F)**.

Arterial endothelial cell eNOS expression was similar between lean (*n* = 14) and obese (*n* = 7) adults (Figures 6A–C). Phosphorylated eNOS (p-eNOS) expression was also similar between lean (*n* = 16) and obese (*n* = 5) adults (Figures 6D–F). Sample sizes are smaller than drug studies due to technical challenges of harvesting an adequate number of cells and because subjects declined the procedure.

## Discussion

We hypothesized young, otherwise healthy obese adults would exhibit preserved EDD with subclinical alterations in NOS and COX signaling. The main findings of this *in vivo* mechanistic human study were as follows. First, we confirm results from our earlier study that EDD is preserved in young (~30 year), obese adults prior to the development of additional clinical-grade cardiovascular risk factors. Second, the functional contribution of NOS to endothelial stimulation is abolished in obese adults. Third, obese humans demonstrate preserved eNOS and p-eNOS expression in arterial ECs. Fourth, the contribution of COX appears similar between the groups. Current results indicate early microvascular alterations in human obesity compensate for a *functional* loss of NOS to preserve EDD, disguising early mechanistic changes. The clinically undetectable reduction in NOS activity may contribute to the onset of cardiovascular disease. Findings provide microvascular and cellular insight to encourage strategies to minimize or reverse the negative vascular impact of human obesity.

### Endothelium-dependent dilation in obesity

Current findings strengthen the concept of preserved microvascular EDD in younger, obese adults (Figure 1; Limberg et al., 2013). Our data are consistent with observations in obese men of similar age (Nielsen et al., 2004) and extend the observation to obese women. In contrast, several studies support a decrement in ACh-mediated vascular responses in obese adults (Steinberg et al., 1996; Perticone et al., 2001; Van Guilder et al., 2006a, 2008; Weil et al., 2011). The discrepancy may be explained by differences in subject age, vascular bed studied, or methods used to quantify blood flow. We focused on adults under 40 years old in the present study, whereas select studies were conducted in adults ranging from 40 to 71 years old (Perticone et al., 2001; Van Guilder et al., 2006a, 2008; Weil et al., 2011). In relatively younger adults (~35 year), Steinberg et al. reported endothelial dysfunction in the leg of obese adults. This suggests early-life vascular changes with obesity might be circulation specific (Steinberg et al., 1996). Perhaps the additional 8 years of aging beyond the current subjects contributed to dysfunction. Alternatively leg blood flow was expressed as a percent increase, such that the 18% larger basal blood flow in obese men created an underestimation of dilation for a given absolute increase in blood flow (Steinberg et al., 1996). Taken in context with data from older obese adults and previously published data from our lab (Limberg et al., 2013), present findings emphasize forearm microvascular EDD is preserved in young, obese adults.

### Role of NOS

Despite preserved EDD in obese adults, *l*-NMMA infusion caused a group difference in ΔFVC (Figure 2). This suggests obese adults have lost the capacity to activate nitric oxide-mediated dilation. The increase in baseline MAP with *l*-NMMA infusion observed in lean adults, but not obese, further supports the loss of NOS in obese adults (Supplementary Table 1). Reduced NOS signaling has previously explained reduced EDD in hypertension (Taddei et al., 1998), healthy aging (Taddei et al., 2001), and diabetes (Huang et al., 2012), but not obesity (Nielsen et al., 2004). The obese adults in the Nielsen et al. study were older, on average, and some obese adults had a BMI < 30 kg m^−2^. Van Guilder et al. also reported obesity does not decrease NOS contribution to ACh-dilation in middle-aged adults (Van Guilder et al., 2008), but the older lean adults in the study may have lost some NOS function with healthy aging (Taddei et al., 2001), making a small difference exceedingly difficult to detect. Our functional EDD data, combined with similar brachial artery eNOS and p-eNOS protein expression between groups, indicate eNOS is functionally dissociated from endothelial stimulation in young, obese adults. These findings are consistent with the idea that a compensatory pathway is upregulated in obesity, masking the functional loss of NOS.

A potential alternate NOS-independent signal in obese subjects is generation of hydrogen peroxide, a known endothelium-dependent hyperpolarizing factor. eNOS is capable of producing substantial vasoactive hydrogen peroxide in response to ACh (Matoba et al., 2000) that is insensitive to NOS inhibition (Yada et al., 2003). ACh-mediated dilation in obese rats is insensitive to NOS inhibition, but is largely inhibited by buffering hydrogen peroxide (catalase; Focardi et al., 2013). This establishes hydrogen peroxide as an alternative vasodilatory mechanism in obesity. In middle-aged and older humans, venous endothelial cell catalase expression is strongly correlated with adiposity, consistent with excess superoxide production during aging or disease progression (Silver et al., 2007). However, we recently demonstrated buffering reactive oxygen species with ascorbic acid infusion did not reduce EDD in younger obese adults (Limberg et al., 2013). While these findings might be explained by ascorbic acid infusion restoring 7,8-dihydrobiopterin (BH_2_) to tetrahydrobiopterin (BH_4_) and recoupling eNOS (Huang et al., 2000), an alternative explanation is an upregulation of endothelium-derived hyperpolarizing factor in younger obese adults independent of reactive oxygen species. Infusion of a cytochrome P450 antagonist reduces basal forearm blood flow in young adults (Spilk et al., 2013). Further, this mechanism is operative when NOS is intact, but may become more important when NOS is impaired (Durand and Gutterman, 2013; Spilk et al., 2013). Specifically, NOS can be lost in obese rats (Chadha et al., 2010) or metabolic syndrome humans (Vigili de Kreutzenberg et al., 2003), while EDHF is upregulated to compensate (Chadha et al., 2010). Future studies aimed at investigating alternate vasodilatory mechanisms will be challenging, due to safety and regulatory concerns regarding pharmacologic tools available for infusion into humans.

### Role of COX

Based on literature, COX signaling is able to compensate when NOS signaling is inhibited (Osanai et al., 2000; Dinenno and Joyner, 2004). Our data support the notion COX does not contribute to ACh-mediated responses in healthy adults (Taddei et al., 1998; Perticone et al., 2001; Schrage et al., 2005). Contrary to our hypothesis, COX inhibition did not create a group difference in ΔFVC to ACh (Figure 3). Previous studies indicate COX limits ACh responses in isolated visceral adipose tissue vessels (Farb et al., 2014) and the forearm of middle-aged (40 year) obese adults (Perticone et al., 2001). Differences may be due to the relatively young age of current subjects, vascular bed studied, or method of COX inhibition. Thus, the contribution of COX to ACh-mediated vasodilation is minimal and unaltered by obesity in younger adults. Further, evidence supports that COX is not the compensatory signal for preserved EDD in young, obese adults.

### Combined NOS-COX inhibition

We investigated the effect of combined NOS-COX inhibition on EDD. Combined inhibition did not create a group difference in ACh responses, except at the 4 μg 100 g^−1^ min^−1^ dose of ACh (Figure 4). Given the group difference with *l*-NMMA alone and no difference with Keto alone, we expected there to be a difference between groups with a similar pattern to *l*-NMMA alone (Figure 2). However, we propose the significant difference at 4 μg 100 g^−1^ min^−1^ and the larger quantitative difference at 16 μg 100 g^−1^ min^−1^ are physiologically relevant (Figure 4). Taking all data into consideration, it appears obesity primarily shifts ECs away from NOS signaling.

### Endothelium-independent dilation

Evidence suggests adiposity may reduce vascular smooth muscle sensitivity to nitric oxide (Christou et al., 2012). However, our data are consistent with previous research in our lab (Limberg et al., 2013) and the body of literature demonstrating preserved vascular smooth muscle function in the arm (Perticone et al., 2001; Nielsen et al., 2004; Van Guilder et al., 2006a, 2008; Weil et al., 2011) and leg (Steinberg et al., 1996) of obese adults. Further, combined NOS-COX inhibition did not create group differences in ΔFVC to NTP (Figure 5). Though data are sparse regarding combined NOS-COX inhibition in young obese adults, our data coincide with research demonstrating NOS-COX inhibition has no effect on NTP responses in young healthy adults (Schrage et al., 2005), and NOS inhibition alone (*l*-NMMA) does not affect NTP responses differently in younger obese adults (Nielsen et al., 2004). Similar responses to NTP allow for clear interpretation of EDD in obesity.

### Etiology of altered endothelial signaling

Although we provide novel evidence of preserved EDD along with preserved eNOS and p-eNOS protein expression in arterial cells, we did not determine the physiologic cause of decreased contribution of NOS to EDD in young, obese adults. Though biomarkers of obesity are beyond the scope of the current aims, we speculate younger obese adults exhibit low-grade inflammation (Van Guilder et al., 2006b), which disrupts endothelial function (Donato et al., 2012). It is also feasible obese adults display early signs of decreased vascular insulin sensitivity. Insulin typically stimulates nitric oxide production (Steinberg et al., 1994). The obese group exhibited twice the fasting plasma insulin concentration of the lean group (Table 1) with lower contribution of NOS (Figure 2). Perhaps chronically elevated insulin reduces the contribution of NOS to EDD (Duncan et al., 2008).

Though mainly involved in metabolism, leptin can cause deleterious effects on the endothelium when in excess (Schinzari et al., 2013). Plasma leptin concentration was over seven times higher in the obese subjects (subset of subjects; 21 obese: 60 ± 35, 18 lean: 8 ± 5 ng mL^−1^). Given the negative effects insulin, leptin, and adiposity may have on vascular function, we correlated these three values to ΔFVC and the change in ΔFVC with *l*-NMMA (absolute and relative). None were significant (data not shown). The absence of significant correlations is expected given the preclinical level of these specific risk factors. On average the obese subjects had significantly greater MAP and triglycerides, and lower HDL cholesterol. None of these three variables reached a level considered to be a clinical risk factor for cardiovascular disease (Pescatello and American College of Sports Medicine, 2014). Discerning a definitive cause of altered NOS signaling is beyond the scope of the current aims; however, this study emphasizes that preclinical endothelial changes are difficult to detect in humans, particularly when overall EDD presents as “normal.” Our data support the hypothesis that excess adiposity combined with chronically elevated insulin and leptin reduce NOS signaling in the face of preserved eNOS and p-eNOS protein expression.

### Experimental considerations

The obese adults exhibited significantly larger forearms, but similar lean forearm mass (Table 1). We dosed ACh and NTP relative to lean forearm mass, not total forearm volume (Taddei et al., 2001; Schrage et al., 2005). We propose this is the most appropriate method of agonist dosing for between-group comparisons as muscle blood flow at rest is much greater than adipose blood flow (Heinonen et al., 2011), such that the vast majority of infused drugs are delivered to the lean tissue. Surprising, we had a less robust effect of *l*-NMMA compared to other studies; however, our dosing of *l*-NMMA is expected to be sufficient, as it was equal to or greater than previously reported (Dinenno and Joyner, 2003; Schrage et al., 2004; Van Guilder et al., 2008; Casey and Joyner, 2009). The difference in *l*-NMMA effects speaks to the variability in NOS signaling in individuals.

Current findings confirm previous data of preserved EDD in young, obese adults (Limberg et al., 2013), yielding greater confidence in our results. Another strength of the current study was the use of *l*-NMMA and Keto individually, in combination, and in reverse order. Though our design did not enable identification of compensatory mechanism(s) that replace NOS signaling in obese adults, current results rule out COX. Furthermore, recent findings from our lab do not support reactive oxygen species as a compensatory vasodilator (Limberg et al., 2013). Future studies will consider inhibiting alternate hyperpolarizing mechanisms (e.g., potassium channels, cytochrome P450).

## Conclusion

Endothelial dysfunction is considered an early marker of overt cardiovascular disease, but the time course of onset and progression of vascular dysfunction has yet to be fully determined. This study contributes to our understanding of the advancement of obesity toward clinical-grade cardiovascular disease. Data demonstrate younger, obese adults retain microvascular endothelial function despite reduced contribution of nitric oxide. Importantly, ECs retain normal eNOS and p-eNOS protein expression, but obesity leads to functional separation of endothelial stimulation and NOS signaling. Data further indicate obesity induces microvascular changes in early adulthood to maintain EDD prior to manifestation of endothelial dysfunction and overt cardiovascular disease. We speculate an early-life loss of NOS signaling contributes to systemic inflammation and when it is combined with advancing age and/or duration of obesity, it becomes the culprit of endothelial dysfunction and overt cardiovascular disease.

## Author contributions

All experiments were conducted in the Bruno Balke Biodynamics Laboratory at the University of Wisconsin–Madison. WS designed the study. JH, RJ, TE, and WS were responsible for data collection, analysis, and interpretation, and manuscript preparation. JS, BW, and ME were involved in study design, medical supervision, and consultation. RS was involved in statistical analysis. All authors approved the final version of this manuscript.

## Funding

Studies were supported by the NIH [HL105820]. Support was provided by the Clinical and Translational Science Award program through the NIH National Center for Advancing Translational Sciences [UL1TR000427] (insulin assay).

### Conflict of interest statement

The authors declare that the research was conducted in the absence of any commercial or financial relationships that could be construed as a potential conflict of interest.

## References

[B1] Acree L. S. Comp P. C. Whitsett T. L. Montgomery P. S. Nickel K. J. Fjeldstad A. S.. (2007). The influence of obesity on calf blood flow and vascular reactivity in older adults. Dyn. Med. 6:4. 10.1186/1476-5918-6-4 17386093 PMC1852303

[B2] Casey D. P. Joyner M. J. (2009). NOS inhibition blunts and delays the compensatory dilation in hypoperfused contracting human muscles. J. Appl. Physiol. (1985) 107, 1685–1692. 10.1152/japplphysiol.00680.2009 19729589 PMC2793197

[B3] Chadha P. S. Haddock R. E. Howitt L. Morris M. J. Murphy T. V. Grayson T. H.. (2010). Obesity up-regulates intermediate conductance calcium-activated potassium channels and myoendothelial gap junctions to maintain endothelial vasodilator function. J. Pharmacol. Exp. Ther. 335, 284–293. 10.1124/jpet.110.167593 20671071

[B4] Christou D. D. Pierce G. L. Walker A. E. Hwang M. H. Yoo J. K. Luttrell M.. (2012). Vascular smooth muscle responsiveness to nitric oxide is reduced in healthy adults with increased adiposity. Am. J. Physiol. Heart Circ. Physiol. 303, H743–H750. 10.1152/ajpheart.00394.2012 22821988 PMC3468458

[B5] Colombo P. C. Ashton A. W. Celaj S. Talreja A. Banchs J. E. Dubois N. B.. (2002). Biopsy coupled to quantitative immunofluorescence: a new method to study the human vascular endothelium. J. Appl. Physiol. (1985) 92, 1331–1338. 10.1152/japplphysiol.00680.2001 11842075

[B6] Davignon J. Ganz P. (2004). Role of endothelial dysfunction in atherosclerosis. Circulation 109, III27–III32. 10.1161/01.CIR.0000131515.03336.f8 15198963

[B7] Delp M. D. Evans M. V. Duan C. (1998). Effects of aging on cardiac output, regional blood flow, and body composition in Fischer-344 rats. J. Appl. Physiol. (1985) 85, 1813–1822. 9804586 10.1152/jappl.1998.85.5.1813

[B8] Dinenno F. A. Joyner M. J. (2003). Blunted sympathetic vasoconstriction in contracting skeletal muscle of healthy humans: is nitric oxide obligatory? J. Physiol. 553, 281–292. 10.1113/jphysiol.2003.049940 12949223 PMC2343482

[B9] Dinenno F. A. Joyner M. J. (2004). Combined NO and PG inhibition augments alpha-adrenergic vasoconstriction in contracting human skeletal muscle. Am. J. Physiol. Heart Circ. Physiol. 287, H2576–H2584. 10.1152/ajpheart.00621.2004 15271659

[B10] Donato A. J. Eskurza I. Silver A. E. Levy A. S. Pierce G. L. Gates P. E.. (2007). Direct evidence of endothelial oxidative stress with aging in humans: relation to impaired endothelium-dependent dilation and upregulation of nuclear factor-kappaB. Circ. Res. 100, 1659–1666. 10.1161/01.RES.0000269183.13937.e8 17478731

[B11] Donato A. J. Henson G. D. Morgan R. G. Enz R. A. Walker A. E. Lesniewski L. A. (2012). TNF-alpha impairs endothelial function in adipose tissue resistance arteries of mice with diet-induced obesity. Am. J. Physiol. Heart Circ. Physiol. 303, H672–H679. 10.1152/ajpheart.00271.2012 22821989 PMC3468456

[B12] Duncan E. R. Crossey P. A. Walker S. Anilkumar N. Poston L. Douglas G.. (2008). Effect of endothelium-specific insulin resistance on endothelial function *in vivo*. Diabetes 57, 3307–3314. 10.2337/db07-1111 18835939 PMC2584137

[B13] Durand M. J. Gutterman D. D. (2013). Diversity in mechanisms of endothelium-dependent vasodilation in health and disease. Microcirculation 20, 239–247. 10.1111/micc.12040 23311975 PMC3625248

[B14] Farb M. G. Tiwari S. Karki S. Ngo D. T. Carmine B. Hess D. T.. (2014). Cyclooxygenase inhibition improves endothelial vasomotor dysfunction of visceral adipose arterioles in human obesity. Obesity (Silver Spring) 22, 349–355. 10.1002/oby.20505 23640904 PMC3766380

[B15] Fildes A. Charlton J. Rudisill C. Littlejohns P. Prevost A. T. Gulliford M. C. (2015). Probability of an obese person attaining normal body weight: cohort study using electronic health records. Am. J. Public Health 105, e54–e59. 10.2105/AJPH.2015.302773 26180980 PMC4539812

[B16] Focardi M. Picchi A. Donnini S. Cameli M. Ziche M. Marzilli M.. (2013). Hydrogen peroxide mediates endothelium-dependent dilation of coronary arterioles in obese rats on a low-carbohydrate diet. Microcirculation 20, 599–608. 10.1111/micc.12058 23517298

[B17] Heinonen I. Bucci M. Kemppainen J. Knuuti J. Nuutila P. Boushel R.. (2012). Regulation of subcutaneous adipose tissue blood flow during exercise in humans. J. Appl. Physiol. (1985) 112, 1059–1063. 10.1152/japplphysiol.00732.2011 22223450

[B18] Heinonen I. Saltin B. Kemppainen J. Sipilä H. T. Oikonen V. Nuutila P.. (2011). Skeletal muscle blood flow and oxygen uptake at rest and during exercise in humans: a pet study with nitric oxide and cyclooxygenase inhibition. Am. J. Physiol. Heart Circ. Physiol. 300, H1510–H1517. 10.1152/ajpheart.00996.2010 21257921

[B19] Herr M. D. Hogeman C. S. Koch D. W. Krishnan A. Momen A. Leuenberger U. A. (2010). A real-time device for converting Doppler ultrasound audio signals into fluid flow velocity. Am. J. Physiol. Heart Circ. Physiol. 298, H1626–H1632. 10.1152/ajpheart.00713.2009 20173048 PMC2867441

[B20] Huang A. Vita J. A. Venema R. C. Keaney J. F. Jr. (2000). Ascorbic acid enhances endothelial nitric-oxide synthase activity by increasing intracellular tetrahydrobiopterin. J. Biol. Chem. 275, 17399–17406. 10.1074/jbc.M002248200 10749876

[B21] Huang A. Yang Y. M. Feher A. Bagi Z. Kaley G. Sun D. (2012). Exacerbation of endothelial dysfunction during the progression of diabetes: role of oxidative stress. Am. J. Physiol. Regul. Integr. Comp. Physiol. 302, R674–R681. 10.1152/ajpregu.00699.2011 22262308 PMC3774487

[B22] Kirby B. S. Voyles W. F. Simpson C. B. Carlson R. E. Schrage W. G. Dinenno F. A. (2009). Endothelium-dependent vasodilatation and exercise hyperaemia in ageing humans: impact of acute ascorbic acid administration. J. Physiol. 587, 1989–2003. 10.1113/jphysiol.2008.167320 19307300 PMC2689338

[B23] Landmesser U. Hornig B. Drexler H. (2004). Endothelial function: a critical determinant in atherosclerosis? Circulation 109, II27–II33. 10.1161/01.CIR.0000129501.88485.1f 15173060

[B24] Limberg J. K. Harrell J. W. Johansson R. E. Eldridge M. W. Proctor L. T. Sebranek J. J.. (2013). Microvascular function in younger adults with obesity and metabolic syndrome: role of oxidative stress. Am. J. Physiol. Heart Circ. Physiol. 305, H1230–H1237. 10.1152/ajpheart.00291.2013 23934859 PMC3798789

[B25] Matoba T. Shimokawa H. Nakashima M. Hirakawa Y. Mukai Y. Hirano K.. (2000). Hydrogen peroxide is an endothelium-derived hyperpolarizing factor in mice. J. Clin. Invest. 106, 1521–1530. 10.1172/JCI10506 11120759 PMC387255

[B26] Nielsen S. Halliwill J. R. Joyner M. J. Jensen M. D. (2004). Vascular response to angiotensin II in upper body obesity. Hypertension 44, 435–441. 10.1161/01.HYP.0000142111.67601.6b 15337733

[B27] Osanai T. Fujita N. Fujiwara N. Nakano T. Takahashi K. Guan W.. (2000). Cross talk of shear-induced production of prostacyclin and nitric oxide in endothelial cells. Am. J. Physiol. Heart Circ. Physiol. 278, H233–H238. 10644603 10.1152/ajpheart.2000.278.1.H233

[B28] Perticone F. Ceravolo R. Candigliota M. Ventura G. Iacopino S. Sinopoli F.. (2001). Obesity and body fat distribution induce endothelial dysfunction by oxidative stress: protective effect of vitamin C. Diabetes 50, 159–165. 10.2337/diabetes.50.1.159 11147782

[B29] Pescatello L. S. American College of Sports Medicine (2014). ACSM's Guidelines for Exercise Testing and Prescription. Philadelphia, PA: Wolters Kluwer/Lippincott Williams & Wilkins Health.

[B30] Pierce G. L. Lesniewski L. A. Lawson B. R. Beske S. D. Seals D. R. (2009). Nuclear factor-{kappa}B activation contributes to vascular endothelial dysfunction via oxidative stress in overweight/obese middle-aged and older humans. Circulation 119, 1284–1292. 10.1161/CIRCULATIONAHA.108.804294 19237660 PMC2810548

[B31] Schinzari F. Tesauro M. Rovella V. Di Daniele N. Mores N. Veneziani A.. (2013). Leptin stimulates both endothelin-1 and nitric oxide activity in lean subjects but not in patients with obesity-related metabolic syndrome. J. Clin. Endocrinol. Metab. 98, 1235–1241. 10.1210/jc.2012-3424 23372172

[B32] Schrage W. G. Dietz N. M. Eisenach J. H. Joyner M. J. (2005). Agonist-dependent variablity of contributions of nitric oxide and prostaglandins in human skeletal muscle. J. Appl. Physiol. (1985) 98, 1251–1257. 10.1152/japplphysiol.00966.2004 15563630

[B33] Schrage W. G. Joyner M. J. Dinenno F. A. (2004). Local inhibition of nitric oxide and prostaglandins independently reduces forearm exercise hyperaemia in humans. J. Physiol. 557, 599–611. 10.1113/jphysiol.2004.061283 15047770 PMC1665102

[B34] Serdula M. K. Ivery D. Coates R. J. Freedman D. S. Williamson D. F. Byers T. (1993). Do obese children become obese adults? A review of the literature. Prev. Med. 22, 167–177. 10.1006/pmed.1993.1014 8483856

[B35] Silver A. E. Beske S. D. Christou D. D. Donato A. J. Moreau K. L. Eskurza I.. (2007). Overweight and obese humans demonstrate increased vascular endothelial NAD(P)H oxidase-p47(phox) expression and evidence of endothelial oxidative stress. Circulation 115, 627–637. 10.1161/CIRCULATIONAHA.106.657486 17242275

[B36] Spilk S. Herr M. D. Sinoway L. I. Leuenberger U. A. (2013). Endothelium-derived hyperpolarizing factor contributes to hypoxia-induced skeletal muscle vasodilation in humans. Am. J. Physiol. Heart Circ. Physiol. 305, H1639–H1645. 10.1152/ajpheart.00073.2013 24043253 PMC3882468

[B37] Steinberg H. O. Brechtel G. Johnson A. Fineberg N. Baron A. D. (1994). Insulin-mediated skeletal muscle vasodilation is nitric oxide dependent. A novel action of insulin to increase nitric oxide release. J. Clin. Invest. 94, 1172–1179. 10.1172/JCI117433 8083357 PMC295191

[B38] Steinberg H. O. Chaker H. Leaming R. Johnson A. Brechtel G. Baron A. D. (1996). Obesity/insulin resistance is associated with endothelial dysfunction. Implications for the syndrome of insulin resistance. J. Clin. Invest. 97, 2601–2610. 10.1172/JCI118709 8647954 PMC507347

[B39] Taddei S. Virdis A. Ghiadoni L. Magagna A. Salvetti A. (1998). Vitamin C improves endothelium-dependent vasodilation by restoring nitric oxide activity in essential hypertension. Circulation 97, 2222–2229. 10.1161/01.CIR.97.22.2222 9631871

[B40] Taddei S. Virdis A. Ghiadoni L. Salvetti G. Bernini G. Magagna A.. (2001). Age-related reduction of NO availability and oxidative stress in humans. Hypertension 38, 274–279. 10.1161/01.HYP.38.2.274 11509489

[B41] Van Guilder G. P. Hoetzer G. L. Dengel D. R. Stauffer B. L. DeSouza C. A. (2006a). Impaired endothelium-dependent vasodilation in normotensive and normoglycemic obese adult humans. J. Cardiovasc. Pharmacol. 47, 310–313. 10.1097/01.fjc.0000205097.29946.d3 16495771

[B42] Van Guilder G. P. Hoetzer G. L. Greiner J. J. Stauffer B. L. Desouza C. A. (2006b). Influence of metabolic syndrome on biomarkers of oxidative stress and inflammation in obese adults. Obesity (Silver Spring) 14, 2127–2131. 10.1038/oby.2006.248 17189537

[B43] Van Guilder G. P. Stauffer B. L. Greiner J. J. Desouza C. A. (2008). Impaired endothelium-dependent vasodilation in overweight and obese adult humans is not limited to muscarinic receptor agonists. Am. J. Physiol. Heart Circ. Physiol. 294, H1685–H1692. 10.1152/ajpheart.01281.2007 18281379 PMC3686114

[B44] Vigili de Kreutzenberg S. Kiwanuka E. Tiengo A. Avogaro A. (2003). Visceral obesity is characterized by impaired nitric oxide-independent vasodilation. Eur. Heart J. 24, 1210–1215. 10.1016/S0195-668X(03)00206-9 12831815

[B45] Weil B. R. Westby C. M. Van Guilder G. P. Greiner J. J. Stauffer B. L. DeSouza C. A. (2011). Enhanced endothelin-1 system activity with overweight and obesity. Am. J. Physiol. Heart Circ. Physiol. 301, H689–H695. 10.1152/ajpheart.00206.2011 21666117 PMC3191085

[B46] Yada T. Shimokawa H. Hiramatsu O. Kajita T. Shigeto F. Goto M.. (2003). Hydrogen peroxide, an endogenous endothelium-derived hyperpolarizing factor, plays an important role in coronary autoregulation *in vivo*. Circulation 107, 1040–1045. 10.1161/01.CIR.0000050145.25589.65 12600919

