# Supplementary material for: Preserved Microvascular Endothelial Function in Young, Obese Adults with Functional Loss of Nitric Oxide Signaling
Source: Front Physiol. 2015 Dec 22;6:387. doi: 10.3389/fphys.2015.00387 (PMC4686588; doi:10.3389/fphys.2015.00387)
Supplement: Supplementary file 1 [file Table1.DOCX]

**Supplemental Table 1**. *Endothelium-dependent vascular responses to ACh*

|  | **Lean** | | | | **Obese** | | | |
| --- | --- | --- | --- | --- | --- | --- | --- | --- |
|  | *Control* | *l-NMMA* | *Keto* | *Combined* | *Control* | *l-NMMA* | *Keto* | *Combined* |
| MAP (mmHg) |  |  |  |  |  |  |  |  |
| Baseline | 83±1 | 90±2 | 85±1 | 89±1 | 91±1 | 94±2 | 94±2 | 96±1 |
| Low | 82±1 | 90±2 | 84±1 | 88±1 | 90±1 | 93±2 | 93±1 | 94±1 |
| Medium | 81±1 | 88±2 | 80±1 | 86±1 | 88±1 | 91±2 | 91±2 | 92±1 |
| High | 82±1 | 89±2 | 81±1 | 86±1 | 88±1 | 91±2 | 90±2 | 92±1 |
| FBF (mL min^-1^) |  |  |  |  |  |  |  |  |
| Baseline | 51±4 | 44±6 | 54±6 | 44±3 | 77±10 | 53±7 | 74±17 | 61±5 |
| Low | 72±8 | 74±11 | 96±12 | 63±6 | 119±14 | 89±11 | 127±36 | 99±12 |
| Medium | 167±14 | 134±21 | 214±21 | 162±14 | 239±25 | 240±29 | 275±46 | 240±19 |
| High | 281±21 | 226±31 | 334±30 | 308±23 | 372±29 | 367±33 | 466±56 | 428±34 |
| FVC (mL min^­-1^ 100 mmHg^-1^) |  |  |  |  |  |  |  |  |
| Baseline | 61±5 | 49±7 | 64±7 | 49±4 | 86±11 | 57±7 | 79±18 | 63±6 |
| Low | 88±9 | 84±13 | 115±15 | 72±7 | 132±15 | 95±11 | 136±38 | 105±12 |
| Medium | 207±17 | 154±25 | 267±26 | 191±17 | 274±29 | 264±31 | 302±50 | 263±21 |
| High | 344±25 | 258±36 | 414±38 | 363±29 | 424±32 | 407±37 | 517±58 | 469±37 |

Values are means ± SEM. MAP, mean arterial blood pressure; FBF, forearm blood flow; FVC, forearm vascular conductance.
