# Supplementary material for: Preserved Microvascular Endothelial Function in Young, Obese Adults with Functional Loss of Nitric Oxide Signaling
Source: Front Physiol. 2015 Dec 22;6:387. doi: 10.3389/fphys.2015.00387 (PMC4686588; doi:10.3389/fphys.2015.00387)
Supplement: Supplementary file 2 [file Table2.DOCX]

**Supplemental Table 2**. *Endothelium-independent vascular responses to NTP*

|  | **Lean** | |  | **Obese** | |
| --- | --- | --- | --- | --- | --- |
|  | *Control* | *Combined* |  | *Control* | *Combined* |
| MAP (mmHg) |  |  |  |  |  |
| Baseline | 84±1 | 90±1 |  | 91±1 | 96±1 |
| Low | 81±1 | 86±1 |  | 87±1 | 92±1 |
| Medium | 79±1 | 84±1 |  | 86±1 | 90±1 |
| High | 77±1 | 82±1 |  | 86±1 | 89±1 |
| FBF (mL min^-1^) |  |  |  |  |  |
| Baseline | 45±4 | 44±4 |  | 90±9 | 63±8 |
| Low | 129±10 | 162±10 |  | 229±26 | 205±25 |
| Medium | 157±10 | 193±14 |  | 244±23 | 255±28 |
| High | 205±13 | 249±17 |  | 293±28 | 293±26 |
| FVC (mL min^-1^ 100 mmHg^-1^) |  |  |  |  |  |
| Baseline | 54±4 | 49±4 |  | 100±10 | 66±8 |
| Low | 161±13 | 191±14 |  | 263±29 | 223±27 |
| Medium | 201±14 | 234±18 |  | 286±27 | 281±30 |
| High | 270±19 | 308±23 |  | 347±34 | 330±30 |
|  |  |  |  |  |  |

Values are means ± SEM. MAP, mean arterial blood pressure; FBF, forearm blood flow; FVC, forearm vascular conductance.
